# Non-Linear Association between Folate/Vitamin B12 Status and Cognitive Function in Older Adults

**DOI:** 10.3390/nu14122443

**Published:** 2022-06-13

**Authors:** Zhe Ding, Lihui Luo, Shaohui Guo, Qing Shen, Yueying Zheng, Shengmei Zhu

**Affiliations:** Department of Anesthesiology, The First Affiliated Hospital, Zhejiang University School of Medicine, Hangzhou 310003, China; dingzhe_@zju.edu.cn (Z.D.); luolihui1212@163.com (L.L.); guo_shaohui@126.com (S.G.); 15058431027@139.com (Q.S.); 1507128@zju.edu.cn (Y.Z.)

**Keywords:** folate, vitamin B12, cognitive function, older adults, NHANES

## Abstract

Although folate and vitamin B12 status have long been implicated in cognitive function, there is no consensus on the threshold of folate and vitamin B12 for assessing their impacts on cognition. The goal of this study was to detail the association between folate and vitamin B12 with cognitive performance. We analyzed cross-sectional data of older adults (≥60 y; *n* = 2204) from the NHANES (National Health and Nutrition Examination Surveys) cohort from 2011–2014. The restricted cubic spline model was used for describing the associations between serum total folate, RBC folate, 5-methyltetrahydrofolate, and vitamin B12 and the Consortium to Establish a Registry for Alzheimer’s Disease Word Learning (CERAD-WL) and Delayed Recall (CERAD-DR) tests, the Animal Fluency (AF) test, and the Digit Symbol Substitution Test (DSST), respectively. Older adults with a different folate and vitamin B12 status were clustered by artificial intelligence unsupervised learning. The statistically significant non-linear relationships between the markers of folate or vitamin B12 status and cognitive function were found after adjustments for potential confounders. Inverse U-shaped associations between folate/vitamin B12 status and cognitive function were observed, and the estimated breakpoint was described. No statistically significant interaction between vitamin B12 and folate status on cognitive function was observed in the current models. In addition, based on the biochemical examination of these four markers, older adults could be assigned into three clusters representing relatively low, medium, and high folate/vitamin B12 status with significantly different scores on the CERAD-DR and DSST. Low or high folate and vitamin B12 status affected selective domains of cognition, and was associated with suboptimal cognitive test outcomes.

## 1. Introduction

There were, reportedly, 43.8 million people with dementia in 2016, a 117% increase from 1990; dementia is approaching being the fifth leading cause of death globally [1]. It has been estimated that the number of people affected will double every 20 years, reaching 81.1 million by 2040 [2]. As the population ages, the incidence of cognitive impairment and dementia increases, making dementia prevention a significant global public health challenge.

Elevated total homocysteine (tHcy) is thought to be a likely causal risk factor for incident dementia based on the results of numerous previous studies [3] (reviewed by Smith [4]). The folate cycle plays a key role in the synthesis of methionine from homocysteine by methionine synthase [5,6]. In the report by Wald et al. [7], the authors estimated that a vitamin B treatment could result in a 3 μmol/L reduction in tHcy, leading to a reduction of 22% for the risk of dementia. There have been a few clinical trials involving vitamin B supplementation for cognitive decline, including the FACIT trial [8], the VITACOG trial [9], and the FAVORIT trial [10]. The results showed that a folic acid treatment, either alone or in combination with vitamin B6 and vitamin B12 supplements, resulted in cognitive benefits, as evidenced by improved processing speed and memory test scores [8,9]. Moreover, a high-dose vitamin B treatment slowed the shrinkage of the whole brain volume, especially in the specific brain regions related to Alzheimer’s disease (AD) [10]. However, the risks associated with excessive folic acid intake have raised concerns, especially since the folic acid food fortification policy. High folate has been proposed to be detrimental to cognition in older people with low vitamin B12 levels [11,12,13]. Moore et al. showed that participants with high folate and normal B12 were also more likely to have an impaired cognitive performance [14].

Although existing reports from observational studies suggest that low or high folate levels are associated with a cognitive impairment, there is no clear definition of the threshold of folate that affects cognitive function. Therefore, we sought to examine the associations between total serum folate, erythrocyte (RBC) folate, 5-methyltetrahydrofolate (5MeTHF), and vitamin B12 and cognitive function, and to assess the possibility of non-linear associations between folate/vitamin B12 status and cognitive function among older participants from the NHANES 2011–2014 cohort. We also performed a cluster analysis to characterize subgroups with different vitamin status groupings and investigated the association between these subgroups with cognitive function, which may provide a possible strategy for dementia prevention.

## 2. Materials and Methods

### 2.1. Data Sources

The data were collected from the National Health and Nutrition Examination Survey (NHANES), a complex, stratified, multistage probability sample survey of non-institutionalized US civilians. The NHANES data were first collected by participants being interviewed at home and then by visiting a mobile examination center (MEC) for a clinical assessment that included the collection of biological samples for analytical measurements. A physical examination, blood draw, and 24 h dietary recall were conducted. All participants or proxies provided written informed consent. The Research Ethics Review Board of the National Center for Health Statistics (NCHS) approved the survey protocol. A detailed description of the NHANES cohort is available elsewhere [15].

### 2.2. Study Population

We searched the NCHS database and found that cognitive function was only tested in the 2011–2012 and 2013–2014 cycles for the sample population of adults aged 60 years and older (*n* = 3472). Supplementary Figure S1 shows the flow chart of eligible participants in this study. We included subjects with complete data of four cognitive assessments, demographic characteristics, past history, and blood examinations. Individuals were further excluded who had a history of renal insufficiency and a reduced estimated glomerular filtration rate (eGFR < 60) or who had a history of strokes. A total of 2204 NHANES participants were suitable for this study.

### 2.3. Biochemical Methods

Details of pre-blood draw fasting were collected via a questionnaire from the MEC prior to the blood draw. The reported duration of the fasting and dietary supplementation varied (<3 h, 3 to <8 h, or ≥8 h) [12]. Whole blood samples were intravenously collected and then spun down to separate out the serum. Serum folate as well as 5MeTHF was determined by HPLC–tandem MS. Microbiological assays were used to estimate the RBC folate. Serum vitamin B12 was determined by a Roche E-170 vitamin B12 electrochemiluminescence immunoassay. Methylmalonic acid (MMA) was assayed by HPLC–tandem MS. The eGFR was calculated for each individual based on the serum creatinine concentration, age, race, and sex using the Chronic Kidney Disease Epidemiology Collaborative equation. Details on the biochemical methods have been described elsewhere [16,17].

### 2.4. Cognitive Test Battery

The cognitive function evaluation included four tests and was collected in person at the MEC. The sequence of the assessment work was as follows: Consortium to Establish a Registry for Alzheimer’s Disease Word Learning (CERAD-WL) test; Animal Fluency (AF) test; Digit Symbol Substitution test (DSST); and CERAD Delayed Recall (CERAD-DR) test. The tests are described in detail in the NHANES documentation and also in previous studies [12,18]. We have provided a brief description of each test below.

CERAD was designed to identify Alzheimer’s disease by assessing new learning, delayed recall, and the ability to recognize memories [19]. The CERAD-WL comprises three consecutive learning trials and an additional delayed recall trial. In the first three learning trials, the participants were asked to read 10 unrelated words aloud. The participants then immediately recalled as many words as possible. The order of the 10 words was changed for each learning trial; the maximum score was 10. The total CERAD-WL score was a maximum of 30. After the other two cognitive exercises were completed, it took approximately 8–10 min to perform the CERAD-DR. The participants were asked to recall the 10 unrelated words used in the first CERAD-WL trial. As per previous research, a threshold of <17 for CERAD-WL and <5 for CERAD-DR were used to distinguish a potential cognitive impairment from a healthy cognitive function and the lack of a cognitive impairment.

The AF test assesses categorical verbal fluency, a component of executive function. Test scores have been shown to distinguish people with a normal cognitive function from those with a mild cognitive impairment and a more severe cognitive impairment (e.g., Alzheimer’s disease) [20,21]. Participants were asked to name three pieces of clothing prior to the test. Those who could not name three pieces of clothing did not continue with the AF exercise. In one minute, the participants were asked to name as many animals as possible. One point was awarded for each correct animal.

The DSST, a performance module from the Wechsler Adult Intelligence Scale (WAIS III), relies on processing speed, sustained attention, and working memory [22]. Participants had 2 minutes to copy the corresponding symbol into the 133 boxes adjacent to the number. The score was the total number of correct matches. Prior to the test, if the participants could not correctly match the symbols to the numbers on their own during the practice, they did not continue to participate in the test.

### 2.5. Covariates

The following variables were assessed for confounders: age (continuous); sex (male and female); marital status (married, widowed, divorced, separated, never married, and living with a partner); race (Mexican American, Hispanic, Non-Hispanic White, Non-Hispanic Black, and Other); educational levels (<9th grade, 9–11th grade, high-school graduate/GED, a college or AA (Associate’s degree) degree, and college graduate or above); body mass index (BMI, continuous); smoking history (yes and no); drinking history (yes and no); diabetes (yes and no); hypertension (yes and no); hyperlipidemia (yes and no); and dietary folate intake (continuous).

More specifically, smoking history was defined as having smoked at least 100 cigarettes in life; drinking history was defined as having had at least 12 alcoholic drinks per 1 year. Diabetes, hypertension, and hyperlipidemia were defined as having ever been told by a doctor or other health professional that they had diabetes, hypertension, or hyperlipidemia, respectively. Dietary folate intake was measured by the dietary interviewer. In the MEC, an in-person interview was conducted; after 3–10 days, a second interview was conducted on the telephone. Based on the dietary records of each participant, total folate (μg), dietary folic acid (μg), food folate (μg), and total dietary folate (μg, expressed in dietary folate equivalents (DFEs)) were calculated [23]. The data used in our study were derived from the first 24 h of the dietary recall as it preceded the blood collection.

### 2.6. Statistical Analysis

The means and standard deviations were calculated for the continuous variables and the proportion was calculated for categorical variables in each category, substratified by vitamin B12 status. A Student’s *t*-test was used to analyze the continuous features.

Multiple linear regression models adjusted for potential confounders were applied to assess the multivariable associations between the folate concentrations and cognitive function. We used restricted cubic spline models fitted for linear regression models with 3 knots at the 10th, 50th, and 90th percentiles of RBC folate, serum total folate, 5MeTHF, and vitamin B12 concentrations, respectively. In our main analysis, we adjusted the model for age (continuous and square transformed), sex (men or women), race/ethnicity (Non-Hispanic White, Non-Hispanic Black, Mexican American, or other race/ethnicity), marital status (married, widowed, divorced, separated, never married, or living with partner), education status (less than high-school, high-school or general education degree, or more than high-school), smoking (yes or no), drinking (yes or no), hypertension history (yes or no), hyperlipidemia history (yes or no), diabetes history (yes or no), estimated glomerular filtration rate (continuous), and folate-related nutrient intakes (total folate, dietary folic acid, food folate, and DFE as continuous variables). A potential non-linearity was also tested using likelihood ratio tests. Where there was evidence of non-linearity in a dose–response curve, a two-line piecewise linear model with a single change point was estimated by including all possible values for the change point and choosing the value with the highest likelihood.

Subgroup analyses were conducted to examine whether the potential association between the folate concentrations and cognitive function was moderated by the vitamin B12 status. According to previous studies [24,25], a vitamin B12 deficiency was classified as <148 pmol/L and elevated MMA was defined as >210 nmol/L. In this study, a low vitamin B12 status was defined as having a low serum B12, an elevated MMA, or both [12]. The group of subjects was divided into a low and normal vitamin B12 group and the above analysis was repeated to ensure the stability of the results. The *p*-values for interaction were evaluated using interaction terms and likelihood ratio tests.

To further detail the association between the folate concentrations and cognitive function, we used K-means clustering to divide the participants into subgroups based on RBC folate, serum total folate, 5MeTHF, and vitamin B12 concentrations. First, we used min–max normalization to scale four factors. In detail, the min–max normalization was performed by applying the following equation so that the values for each variable ranged from 0 to 1:

(1) $$\mathrm{Normalized}\;\mathrm{value}\;\left(0-1\right)=\frac{value-value_{min}}{value_{max}-value_{min}}$$

We then used K-means clustering to group the participants on the basis of the four normalized variables. K-means clustering is a conventional clustering algorithm that has been used to uncover novel subgroups of adult-onset diabetes [26], dietary patterns [27], and vulnerable subpopulations among Medicare patients undergoing a total joint arthroplasty [28]. The effect of clustering for each different value of K was measured by using the Carinski–Harabasz score [29]. To use K-means clustering, it is necessary to determine the hyperparameter K, which is the number of groups or clusters into which the data can be arranged. In this study, the highest sum-of-squares distance and Carinski–Harabasz scores were obtained when K was 3. Therefore, the participants were apportioned into three groups through 10,000 iterations of the K-means algorithm. The clusterwise stability was assessed through resampling the dataset 2000 times and computing the Jaccard similarities to the original cluster. We analyzed the differences in cognitive function among the three different clustering groups by an ANOVA to determine that the classifiers were clinically significant.

All analyses were performed in R programming language software, version 4.1 (R Foundation). Bilateral *p*-values less than 0.05 were defined as statistically significant.

## 3. Results

The baseline characteristics of the study population (*n* = 2204) divided by low (*n* = 635, 28.8%) and normal vitamin B12 (*n* = 1569, 71.2%) are shown in Table 1. Significant differences were present among the participants by age (*p* < 0.001), race/ethnicity (*p* < 0.001), marital status (*p* = 0.002), and education (*p* = 0.023), but not by sex, BMI, alcoholic drinks, smoking, hypertension, hyperlipidemia, diabetes, or nutrient intakes of folate/folic acid and vitamin B12.

**Figure 1:**
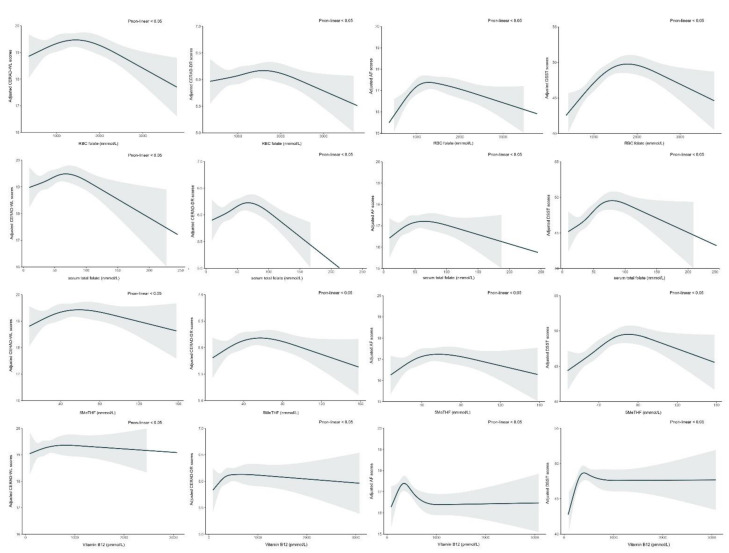
Association between folate/vitamin B12 status and cognitive performance. The associations between the cognitive level and RBC folate, serum total folate, 5MeTHF, and vitamin B12 were assessed by restricted cubic spline curves based on a linear model estimation using ordinary least squares. In Figure 1, the solid black lines with shaded regions indicate the adjusted recognition test scores and their corresponding 95% confidence intervals, respectively.

After adjusting for the potential confounders mentioned in the Material and Methods section, associations between RBC folate, serum total folate, 5MeTHF, and vitamin B12 and cognitive performance—including CERAD-WL, CERAD-DR and AF as well as DSST—were reverse U-shaped (Figure 1). The cognitive scores reached a peak at an RBC folate threshold point, with positive associations below and inverse associations above the change point. A similar shape of non-linear associations was also found when the relation between serum total folate, 5MeTHF, and vitamin B12 and cognitive performance was evaluated (all *p* for non-linear < 0.05). Moreover, it showed that the non-linear associations between the folate status and cognitive function in the presence of a normal vitamin B12 status were significant (Supplementary Figure S2; *p*-value for non-linear < 0.05). In the low vitamin B12 status, the *p*-value for the non-linear relationship between RBC folate and CERAD-WL as well as CERAD-DR was >0.05 and the *p*-value for the non-linear relationship between serum total folate and CERAD-WL was >0.05 (Supplementary Figure S3). In our restricted cubic spline model, the interactions between vitamin B12 and folate status in relation to the cognitive tests were not significant (all *p*-value for interaction > 0.05, Supplementary Table S1).

**Figure 2:**
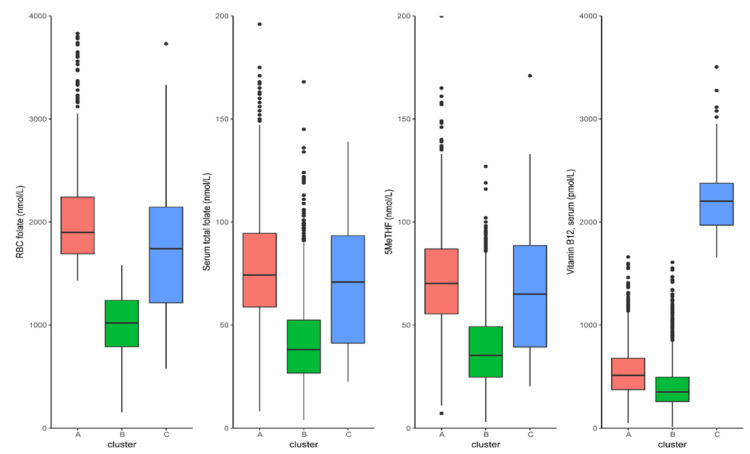
The characterization of cognitive performance in different clusters of older adults. Distribution of RBC folate, serum total folate, 5MeTHF, and vitamin B12 concentrations in the NHANES cohort for each cluster (Cluster A: red, Cluster B: green, Cluster C: blue).

Using the K-means clustering algorithm, three clusters representing the folate and vitamin B12 status (red: Cluster A; green: Cluster B; blue: Cluster C) were identified and are characterized in Figure 2. The dataset was resampled 2000 times and the Jaccard similarity to the original clusters was calculated. The stability of the clusters was estimated as the Jaccard mean, which was greater than 0.8 for all clusters (Supplementary Figure S4). Participants in Cluster B (1493 participants, 67.74%) had the lowest concentration of RBC folate (1016.17 ± 295.11 nmmol/L), serum total folate (41.87 ± 20.59 nmmol/L), 5MeTHF (38.7 ± 18.81 nmmol/L), and vitamin B12 (403.22 ± 216.91 pmmol/L) compared with the other clusters. Cluster C (57 participants, 2.59%) was characterized by a higher concentration of serum total folate (92.11 ± 73.55 nmmol/L), 5MeTHF (81.11 ± 61.89 nmmol/L), and vitamin B12 (2681.36 ± 1285.76 pmmol/L). The older adults in Cluster A (654 participants, 29.67%) were characterized by a relatively intermediate concentration of serum total folate (79.73 ± 35.01 nmmol/L), 5MeTHF (72.49 ± 28.22 nmmol/L), and vitamin B12 (565.37 ± 280.17 pmmol/L) (Table 2). The differences between scores from the CERAD-DR and DSST were significant in all three clusters (Supplementary Table S2; *p* = 0.039 for CERAD-DR, *p* = 0.004 for DSST). Compared with Clusters B and C, Cluster A was characterized by the highest scores for CERAD-DR and DSST. Compared with Cluster B, Cluster C had higher DSST scores and lower CERAD-DR scores. As mentioned in the Materials and Methods section, CERAD-DR is a recall of the words used in the first CERAD-WL trial; the CERAD-WL scores did not significantly differ across the three clusters. There was no difference in the score of the AF test (Supplementary Table S2).

The characterization of older adults according to the three folate and vitamin B12 status is presented in Table 3. No differences between the clusters were found by BMI (*p* = 0.685), alcoholic drinks (*p* = 0.421), diabetes (*p* = 0.709), or taking treatment for anemia (*p* = 0.337). Significant differences were present among the clusters by hypertension (*p* < 0.014) or hyperlipidemia (*p* = 0.003). Cluster B included a lower percentage of women (46.5%) compared with the other two clusters whereas Cluster C included a higher percentage of women (63.2%). With regard to education, higher education (college or college graduate or above) increased the odds of being included in Cluster A (56.6%) whereas it decreased the odds of being included in Cluster B (51.7%). A higher proportion of older adults with an education level less than high-school (12.1%) belonged to Cluster B. Compared with the other two clusters, Cluster B had the highest percentage of older adults who smoked. Regarding dietary supplement use, folic acid and vitamin B12 supplementation were the highest in Cluster A and the lowest in Cluster B.

## 4. Discussion

In this study, the non-linear relationships between folate and vitamin B12 status and cognitive function were investigated. We sought to explore the trend of this inverted U-shaped relationship where lower and higher concentrations affected cognitive function both before and after a threshold, and further explored this trend in the context of a vitamin B12 deficiency. Moreover, we performed clustering and older adults were classified into three subgroups representing relatively low, medium, and high folate and vitamin B12 status. Significant differences in CERAD-DR and DSST scores were present among the three clusters.

Previous observational studies and case reports have observed that excess folic acid is detrimental to cognitive function, and intervention studies have indicated that vitamin B12 and folic acid supplementation is beneficial for certain cognitive functions (as summarized by Smith and Refsum [4] and by Field and Stover [31]). Our results showed that either a too high or a too low folate/vitamin B12 status was associated with the trend of cognitive decline (Figure 2). It is reasonable to believe that a desirable folate and vitamin B12 status is associated with optimal cognitive function. However, the assessment of the folate and vitamin B12 status, i.e., high and low thresholds, remains unclear. Much of the controversy relates to methodological issues. Inter-laboratory and inter-platform methodologies for measuring folate and vitamin B12 have important influences on the accuracy of any cutoff point that is established [32,33].

The issue is how to define a suitable folate and vitamin B12 status among the elderly population. Previous NHANES studies have defined the folate status by population percentile cutoffs or by references to previous literature [12,34]. Our study was a mathematical model to investigate what levels of folate and vitamin B12 were optimal for different cognitive domains, and what the shapes of the associations were if non-linear. In this paper, based on the inverted U-shaped relationship between the folate/vitamin B12 status and cognitive function, we speculated that there may be a concentration range of folate and vitamin B12 within which cognition is optimal. Based on the current restricted cubic spline model, possible breakpoints corresponding with four separate cognitive tests were obtained (Supplementary Table S1). The relationship between the folate/vitamin B12 status and optimal cognition may vary depending on the different cognitive tests, i.e., the concentration ranges of the different biomarkers varied from test to test. Compared with CERAD-WL, CERAD-DR, and AF, the breakpoints of the non-linear curves of the folate status and DSST were shifted to the right. This may be due to the different sensitivity of specific cognitive functions with regard to an excess or a deficiency of folate. Additionally, it should be noted that the breakpoints generated by the current model had the same methodological limitations and will vary by the platform used to measure these vitamins. In addition, a population-based assessment may not provide certainty with regard to the true status of every single individual [32]. More research is needed in the future to help reach a good consensus on appropriate biomarker cutoff points to define the severity of a folate and vitamin B12 deficiency or excess.

There are several potential mechanisms to support the observed associations between folate and cognitive function. Folic acid supplementation is effective in reducing plasma Hcy levels [7]. Hcy is an independent risk factor for AD and vascular dementia, and the risk of AD is doubled with Hcy >14 umol/L [35]. Possible mechanisms include toxic effects on neurons due to vascular factors, an abnormal aggregation of tau protein activation, and the inhibition of methylation responses [4]. Vitamin B12 and folic acid are required for the methionine synthase reaction in which a methyl group is transferred from methyltetrahydrofolate to homocysteine by methionine synthase and methyl-B12 acts as a coenzyme to form methionine [5,6,36]. A vitamin B12 deficiency can lead to the accumulation of 5MeTHF in cells (i.e., the 5MeTHF trap), resulting in a functional folate deficiency and impaired DNA biosynthesis [36].

In the low vitamin B12 status group, the overall inverted U-shaped trends between folate and CERAD were changed (Supplementary Figure S3). A vitamin B12 deficiency is prevalent in the elderly and is associated with cognitive impairment [37,38]. Previous research has indicated that the progressive shrinkage of the brain was associated with a decrease in vitamin B12 and holotranscobalamin (holoTC, served as active vitamin B12) levels [39]. It has also been reported that periventricular white matter lesions (pWML) were associated with low levels of vitamin B12 in plasma [40,41]. Moreover, psychiatric symptoms caused by white matter damage in the brain due to a vitamin B12 deficiency could precede cognitive decline and be alleviated by vitamin B12 supplementation [42,43]. In general, it suggests a close relationship between a vitamin B12 deficiency and a decline in cognition. Table 1 also shows that a vitamin B12 deficiency is associated with a potential cognitive impairment. A low vitamin B12 status may contribute to cognitive decline, independent of folate levels [31]. The mechanisms by which these associations may arise remain unclear and need to be explored in cellular and animal models to further address this question.

Using the NHANES 2011–2014 study [12,34], previous studies have shown insufficient evidence that cognitive deterioration is associated with concurrent high folate and insufficient vitamin B12 concentrations. In our study, the interactions between vitamin B12 and folate status in relation to cognitive tests were not significant by using the restricted cubic spline model (Supplementary Table S1). It has been suggested that people with high folate and low vitamin B12 are at a risk of a poor health status, which may be due to underlying health conditions such as vitamin B12 malabsorption or a renal insufficiency rather than due to their high folate status or folic acid intake [34]. Recent literature has found that people with a cognitive impairment were less likely to use supplements containing folic acid or vitamin B12 than people without a cognitive impairment (mean ± SEE: 34.4% ± 2.4% vs. 47.5% ± 1.6%) [34]. A cognitive impairment may have a casual effect on the vitamin B12 and/or folate status (i.e., reverse causation) or be indirectly or co-related.

Using an artificial intelligence unsupervised learning approach, three clinical clusters were obtained based on the laboratory assessments of three forms of folate and vitamin B12. The clustering showed that, compared with Cluster B and C, older adults in Cluster A obtained the highest scores on all four cognitive tests (Supplementary Table S2). Moreover, serum total folate, 5MeTHF, vitamin B12, MMA, and unmetabolized serum folic acid (UMFA) concentrations in older adults in Cluster A were between Cluster B and C. This was consistent with the findings of a non-linear relationship between folate/vitamin B12 status and cognitive function (Figure 1).

By clustering, the cognitive performance varied among the subgroups with significant differences between the subgroups for the CERAD-DR and DSST scores, but no differences between the three subgroups for the AF test (Supplementary Table S2). A previous large randomized, double-blind, placebo-controlled study found significant improvements in memory, information processing speed, and sensorimotor speed in a 3-year folic acid supplementation group whereas the verbal fluency performance did not differ compared with the control group [8].

Cluster B represented two-thirds of the study population with a low vitamin status (Table 3). Compared with Clusters A and C, Cluster B had the lowest DSST score. Consistent with our study, a recent study also found that high-dose vitamin B supplementation provided benefits in processing speed and memory with a significant increase in DSST scores [9].

Cluster A had the highest DSST score and the highest dietary folic acid and vitamin B12 intake; this group was mainly supplement users. Moreover, compared with B and C, Cluster A had the highest percentage of older adults who were NHW and married, and who had high levels of education (college or above). Marital status and education may affect the vitamin status by influencing different dietary patterns. Individual vitamin status is related to multiple factors such as genetics (race) and dietary and related nutrient intakes, and, therefore, requires a comprehensive evaluation.

Our study had several limitations. Due to the cross-sectional nature of the data, a causal relationship between folate/vitamin B12 status and cognition could not be inferred. Poor folate and vitamin B12 status are proposed as risk factors for age-related cognitive decline, but their causality remains unclear. Moreover, owing to ethical issues, we can only further reveal the mechanisms through cellular and animal studies. The non-linear relationships and clustering explored in this study were based on the US elderly population (2011–2014 NHANES data), which is the country where folic acid fortification is administered; therefore, this limitation needs to be clarified when these results are referenced in other national populations. In addition, although the NHANES collected comprehensive information on demographics, lifestyles, and health status, allowing us to consider known key confounders, there is always the potential for residual confounders.

## Figures and Tables

**Table 1:** Baseline characteristics of the study population.

Characteristics	Participants, No. (%)			*p*-Value
	Total Sample (*N* = 2204)	Vitamin B12 Status		
		Low ^2^ (*N* = 635)	Normal (*N* = 1569)	
Age, y ^1^	69.07 ± 6.70	71.09 ± 6.98	68.26 ± 6.42	<0.001
Female, *n* (%)	1108 (50.3)	318 (50.1)	790 (50.4)	0.945
Race/ethnicity ^3^, *n* (%)				<0.001
MA	202 (9.2)	50 (7.9)	152 (9.7)	
Hispanic	232 (10.5)	60 (9.4)	172 (11.0)	
NHW	1101 (50.0)	401 (63.1)	700 (44.6)	
NHB	480 (21.8)	89 (14.0)	391 (24.9)	
Other	189 (8.6)	35 (5.5)	154 (9.8)	
Marital status, *n* (%)				0.002
Married	1259 (57.1)	327 (51.5)	932 (59.4)	
Widowed	388 (17.6)	144 (22.7)	244 (15.6)	
Divorced	316 (14.3)	91 (14.3)	225 (14.3)	
Separated	53 (2.4)	17 (2.7)	36 (2.3)	
Never married	122 (5.5)	34 (5.4)	88 (5.6)	
Living with partner	66 (3.0)	22 (3.5)	44 (2.8)	
Education, *n* (%)				0.023
Less than 9th grade	230 (10.4)	82 (12.9)	148 (9.4)	
9–11th grade	291 (13.2)	89 (14.0)	202 (12.9)	
High-school graduate/GED	510 (23.1)	155 (24.4)	355 (22.6)	
A college or AA degree	636 (28.9)	177 (27.9)	459 (29.3)	
College graduate or above	537 (24.4)	132 (20.8)	405 (25.8)	
BMI ^3^ (kg/m^2^) ^1^	29.03 ± 6.17	29.23 ± 6.74	28.95 ± 5.93	0.329
Alcoholic drinks, *n* (%)	1542 (70.0)	445 (70.1)	1097 (69.9)	0.799
Smoking, *n* (%)	1093 (49.6)	330 (52.0)	763 (48.6)	0.253
Hypertension, *n* (%)	1310 (59.4)	395 (62.2)	915 (58.3)	0.143
Hyperlipidemia, *n* (%)	1216 (55.2)	338 (53.2)	878 (56.0)	0.228
Diabetes, *n* (%)	464 (21.1)	136 (21.4)	328 (20.9)	0.481
Nutrient Intake				
Total folate (ug)	379.75 ± 232.47	379.17 ± 240.97	379.99 ± 229.02	0.94
Dietary folic acid (ug)	165.93 ± 162.73	172.15 ± 172.88	163.42 ± 158.42	0.254
Food folate (ug)	213.85 ± 145.66	207.06 ± 128.43	216.60 ± 152.03	0.164
Folate, DFE ^3^ (ug) Vitamin B12 (ug) Cognitive assessment ^3,4^	495.92 ± 329.51 0.94 ± 2.29	499.68 ± 350.43 0.84 ± 2.09	494.39 ± 320.76 0.97 ± 2.36	0.733 0.222
WL < 17 and DR < 5, *n* (%)	344 (15.6)	125 (19.7)	219 (14.0)	0.001
AF < 14, *n* (%)	601 (27.3)	196 (30.9)	405 (25.8)	0.018
DSST < 34, *n* (%)	476 (21.6)	167 (26.3)	309 (19.7)	<0.001

^1^ Values are mean ± standard deviations (SD). ^2^ B12 deficiency was classified as <148 pmol/L and elevated MMA was defined as >210 nmol/L. Low vitamin B12 status was operationally defined in this study as having a low serum B12, an elevated MMA, or both. ^3^ MA: Mexican American; NHB: Non-Hispanic Black; NHW: Non-Hispanic White; BMI: body mass index; DFE: dietary folate equivalents; 1 DFE = 1 ug food folate = 0.6 ug folic acid from supplements and fortified food [30]. CERAD-WL: Consortium to Establish a Registry for Alzheimer’s Disease Word Learning; DR: Delayed Recall; AF: Animal Fluency test; DSST: Digit Symbol Substitution Test. ^4^ In this study, a cutoff of <17 for CERAD-WL, <5 for CERAD-DR, <14 for AF, and <34 for DSST was used to define a potential cognitive impairment [12].

**Table 2:** Mean (SD) concentrations of markers representing folate and vitamin B12 status of US older adults (≥60 y; *n* = 2204) from the 2011–2014 NHANES cohort.

Marker	Concentrations				*p*-Value
	Total Sample (*N* = 2204)	Cluster			
		A (*N* = 654)	B (*N* = 1493)	C (*N* = 57)	
RBC folate (nmmol/L)	1353.79 (643.75)	2076.02 (550.81)	1016.17 (295.11)	1910.47 (999.11)	<0.001
Serum total folate (nmmol/L)	54.4 (33.49)	79.73 (35.01)	41.87 (20.59)	92.11 (73.55)	<0.001
5MeTHF (nmmol/L)	49.82 (28.89)	72.49 (28.22)	38.70 (18.81)	81.11 (61.89)	<0.001
Vitamin B12 (pmmol/L)	510.25 (477.27)	565.37 (280.17)	403.22 (216.91)	2681.36 (1285.76)	<0.001
MMA (nmmol/L)	202.16 (162.43)	200.57 (138.48)	205.40 (173.89)	135.39 (61.64)	0.006
UMFA (nmmol/L)	2.74 (9.49)	5.08 (14.37)	1.49 (4.24)	8.80 (22.60)	<0.001

5MeTHF: 5-methyltetrahydrofolate; MMA: methylmalonic acid; UMFA: unmetabolized serum folic acid.

**Table 3:** Characteristics of clusters representing folate and vitamin B12 status of US older adults (≥60 y; *n* = 2204) from the 2011–2014 NHANES cohort.

Characteristics	Participants, No. (%)				*p*-Value
	Total Sample (*N* = 2204)	Cluster			
		A (*N* = 654)	B (*N* = 1493)	C (*N* = 57)	
Female, *n* (%)	1108 (50.3)	378 (57.8)	694 (46.5)	36 (63.2)	<0.001
Age	69.07 ± 6.70	71.11 ± 6.95	68.12 ± 6.38	70.63 ± 6.55	<0.001
Race/ethnicity ^1^, *n* (%)					<0.001
MA	202 (9.2)	29 (4.4)	169 (11.3)	4 (7.0)	
Hispanic	232 (10.5)	51 (7.8)	175 (11.7)	6 (10.5)	
NHW	1101 (50.0)	442 (67.6)	631 (42.3)	28 (49.1)	
NHB	480 (21.8)	79 (12.1)	389 (26.1)	12 (21.1)	
Other	189 (8.6)	53 (8.1)	129 (8.6)	7 (12.3)	
Marital status, *n* (%)					0.016
Married	1259 (57.1)	389 (59.5)	840 (56.3)	30 (52.6)	
Widowed	388 (17.6)	129 (19.7)	245 (16.4)	14 (24.6)	
Divorced	316 (14.3)	83 (12.7)	225 (15.1)	8 (14.0)	
Separated	53 (2.4)	7 (1.1)	43 (2.9)	3 (5.3)	
Never married	122 (5.5)	31 (4.7)	91 (6.1)	0 (0.0)	
Living with partner	66 (3.0)	15 (2.3)	49 (3.3)	2 (3.5)	
Education, *n* (%)					0.001
Less than 9th grade	230 (10.4)	43 (6.6)	181 (12.1)	6 (10.5)	
9–11th grade	291 (13.2)	94 (14.4)	189 (12.7)	8 (14.0)	
High-school graduate/GED	510 (23.1)	147 (22.5)	351 (23.5)	12 (12.1)	
A college or AA degree	636 (28.9)	187 (28.6)	438 (29.3)	11 (19.3)	
College graduate or above	537 (24.4)	183 (28.0)	334 (22.4)	20 (35.1)	
BMI ^1^ (kg/m^2^) ^2^	29.03 ± 6.17	29.11 ± 5.98	29.02 ± 6.28	28.38 ± 5.73	0.685
Alcoholic drinks, *n* (%)	1542 (70.0)	442 (67.6)	1063 (71.2)	37 (64.9)	0.421
Smoking, *n* (%)	1093 (49.6)	296 (45.3)	775 (51.9)	22 (38.6)	0.017
Hypertension, *n* (%)	1310 (59.4)	424 (64.8)	854 (57.2)	32 (56.1)	0.014
Hyperlipidemia, *n* (%)	1216 (55.2)	393 (60.1)	793 (53.1)	30 (52.6)	0.003
Diabetes, *n* (%)	464 (21.1)	142 (21.7)	306 (20.5)	16 (8.1)	0.709
Taking treatment for anemia ^3^, *n* (%)	80 (3.6)	31 (4.7)	48 (3.2)	1 (1.8)	0.337
Nutrient intakes ^2^					
Total folate (ug)	379.75 ± 232.47	410.07 ± 245.23	364.53 ± 214.32	430.67 ± 425.98	<0.001
Dietary folic acid (ug)	165.93 ± 162.73	201.87 ± 195.04	150.60 ± 142.87	155.16 ± 181.36	<0.001
Food folate (ug)	213.85 ± 145.66	208.26 ± 120.57	213.94 ± 136.54	275.46 ± 405.25	0.004
Folate, DFE ^1^ (ug)	495.92 ± 329.51	551.37 (370.67)	469.98 ± 298.94	539.05 ± 482.30	<0.001
Vitamin B12 (ug)	0.94 ± 2.29	1.32 ± 2.50	0.75 ± 2.14	1.32 ± 2.76	<0.001

^1^ MA: Mexican American; NHB: Non-Hispanic Black; NHW: Non-Hispanic White; BMI: body mass index; DFE: dietary folate equivalents. ^2^ Values are mean ± standard deviations (SD). ^3^ Taking treatment for anemia during the past 3 months according to the information collected from the questionnaire by NHANE.

## Data Availability

Data described in the manuscript, code book, and analytic code will be made publicly and freely available without restriction at https://wwwn.cdc.gov/nchs/nhanes/continuousnhanes/labmethods.aspx?BeginYear=2011 (accessed on 10 June 2022), and https://wwwn.cdc.gov/nchs/nhanes/continuousnhanes/labmethods.aspx?BeginYear=2013 (accessed on 5 May 2022). The analytic code used for the manuscript is available upon request from the corresponding author.

## Supplementary Materials

The following are available online at https://www.mdpi.com/article/10.3390/nu14122443/s1, Table S1: Breakpoints of the non-linear association between folate/vitamin B12 status and cognitive performance; Table S2: Mean (SD) scores of each cognitive test according to folate and vitamin B12 status; Figure S1: Flowchart illustrating sample stratification; Figure S2: Association between folate status and cognitive performance in normal vitamin B12 status; Figure S3: Association between folate status and cognitive performance in low vitamin B12 status; Figure S4: Elbow plot and mean silhouette coefficients plot.
