# Supplementary material for: Non-Linear Association between Folate/Vitamin B12 Status and Cognitive Function in Older Adults
Source: Nutrients. 2022 Jun 13;14(12):2443. doi: 10.3390/nu14122443 (PMC9227588; doi:10.3390/nu14122443)
Supplement: Supplementary file 1 [file nutrients-14-02443-s001.zip › nutrients-1742902-supplementary.pdf]

Supplementary Materials

Table S1. Breakpoints of the non-linear association between folate /vitamin B12 status and cognitive performance

Marker	Cognitive tests			
	Memory recall	Memory delayed recall	Animal Fluency	Digit Symbol
RBC folate (nmol/L)				
Overall	1450	1600	1280	1780
[1] Low vitamin B12 status	/	/	1130	1510
Normal vitamin B12 status	1530	1640	1490	1820
p Value for interaction	0.317	0.809	0.291	0.423
Serum total folate (nmol/L)				
Overall	67.4	67.1	64.6	79.3
Low vitamin B12 status	/	67.2	49.3	82.6
Normal vitamin B12 status	50.6	51.7	74.1	77.5
p Value for interaction	0.268	0.075	0.262	0.828
5MeTHF (nmol/L)				
Overall	58.7	57.1	57.1	70.2
Low vitamin B12 status	70.56	60.8	40.85	71.4
Normal vitamin B12 status	/	44.8	66.3	69.6
p Value for interaction	0.172	0.073	0.332	0.764
Vitamin B12 (pmol/L)				
Overall	811.8	659	345.4	405.2

[1]B12 deficiency was classified as < 148 pmol/L, while elevated MMA was defined as > 210 nmol/L. Low vitamin B12 status was operationally defined in this study as having a low serum B12, an elevated MMA, or both.

/ refers to non appliable for estimated breakpoints in non-linear model (*p* for non-linear > 0.05)

5MeTHF: 5-methyltetrahydrofolat

Table S2. Mean (SD) scores of each cognitive test according to folates and vitamin B12 status

Cognitive tests	Test Scores				*P* value
	Total Sample	Cluster			
	(N=2204)	A (N=654)	B (N=1493)	C (N=57)	
CERAD-WL, mean (SD)	19.25 (4.50)	19.16 (4.74)	19.29 (4.39)	19.19 (4.72)	0.837
CERAD-DR, mean (SD)	6.07 (2.29)	6.09 (2.32)	6.07 (2.28)	6.05 (2.46)	0.039
AF, mean (SD)	17.00 (5.47)	16.98 (5.35)	17.01 (5.54)	16.82 (5.17)	0.965
DSST, mean (SD)	47.65 (16.93)	49.42 (16.05)	46.84 (17.29)	48.75 (15.76)	0.004

CERAD-WL/DR: consortium to establish a registry for Alzheimer's disease -word learning/ delayed recall, AF: animal fluency, DSST: digit symbol substitution test

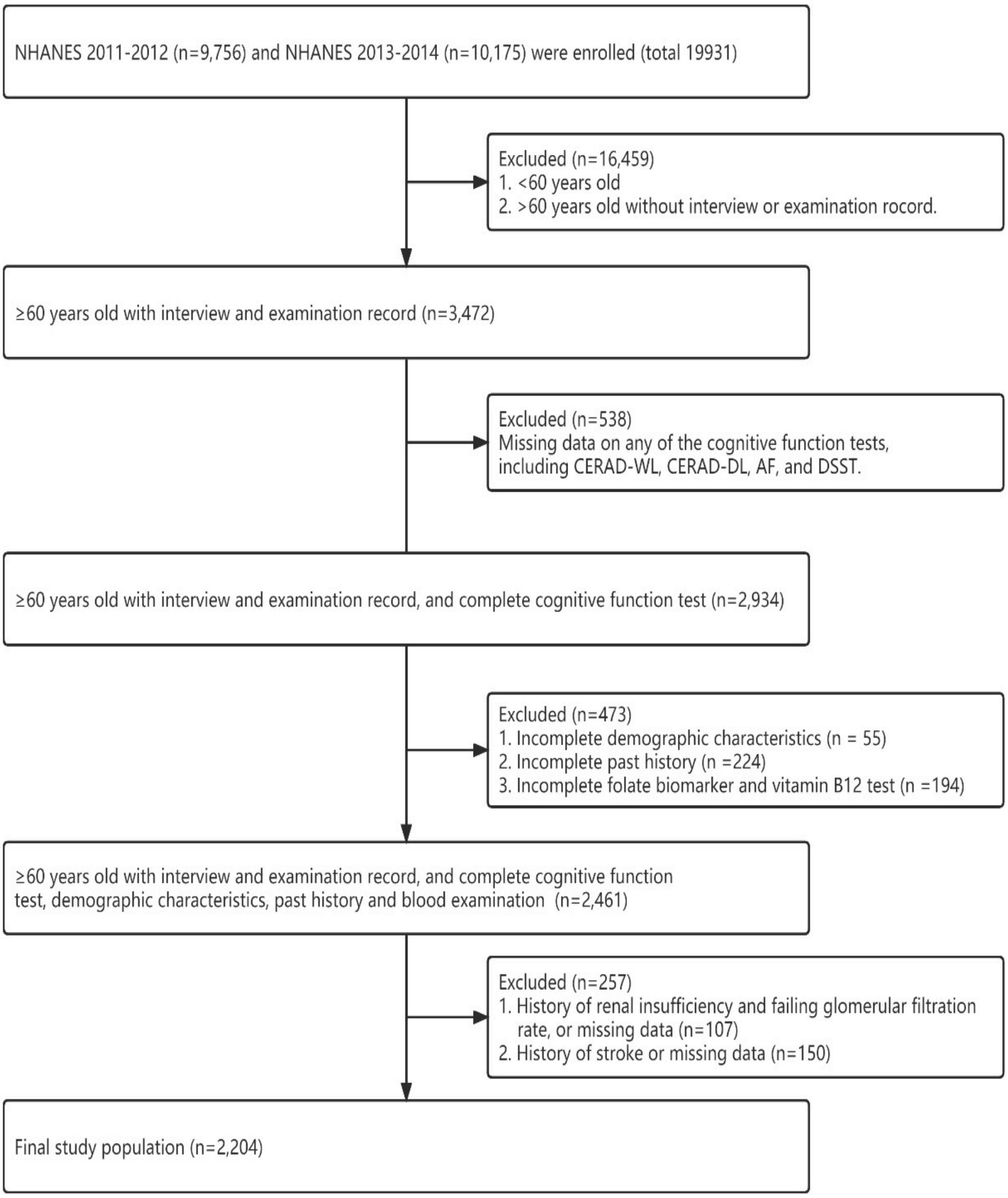

Figure S1. Flowchart illustrating sample stratification.

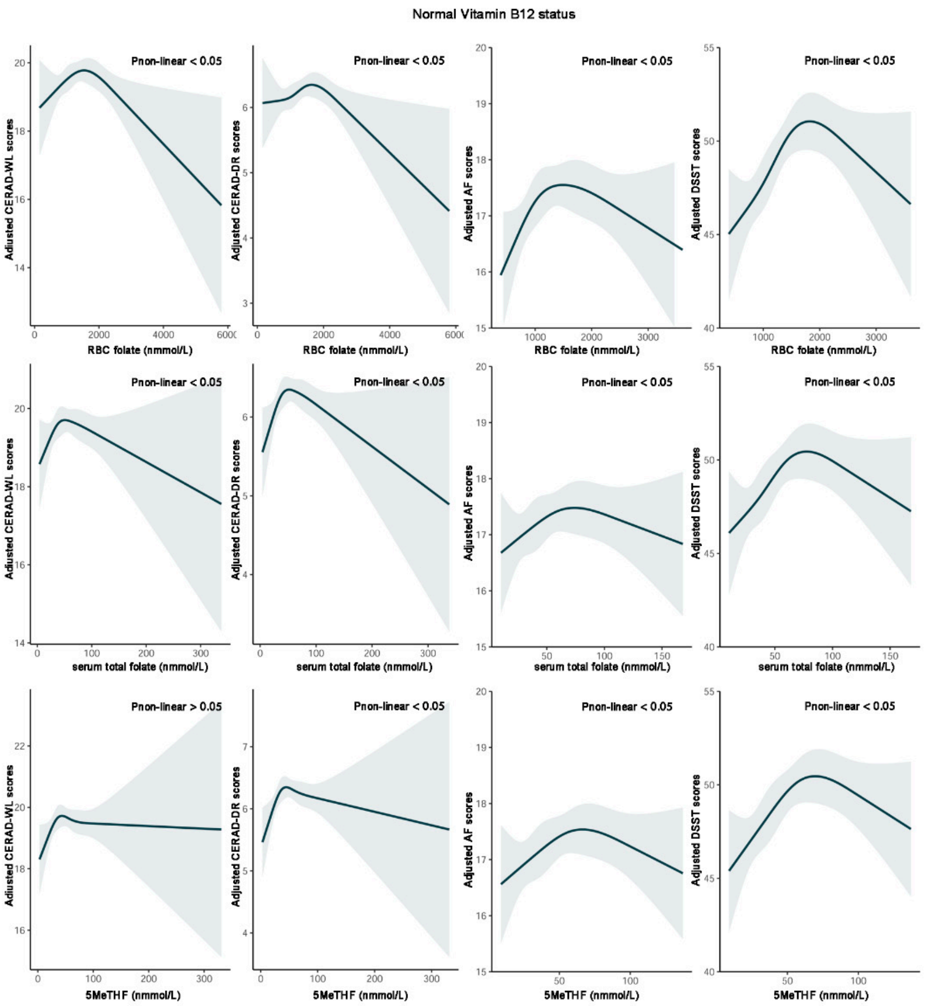

Figure S2. Association between folate status and cognitive performance in normal vitamin B12 status. The association between cognitive level and RBC folate, serum total folate, 5MeTHF was assessed by restricted cubic spline curves based on linear model estimation using ordinary least squares. The solid black lines with shaded regions indicate the adjusted recognition test scores and their corresponding 95% confidence intervals, respectively.

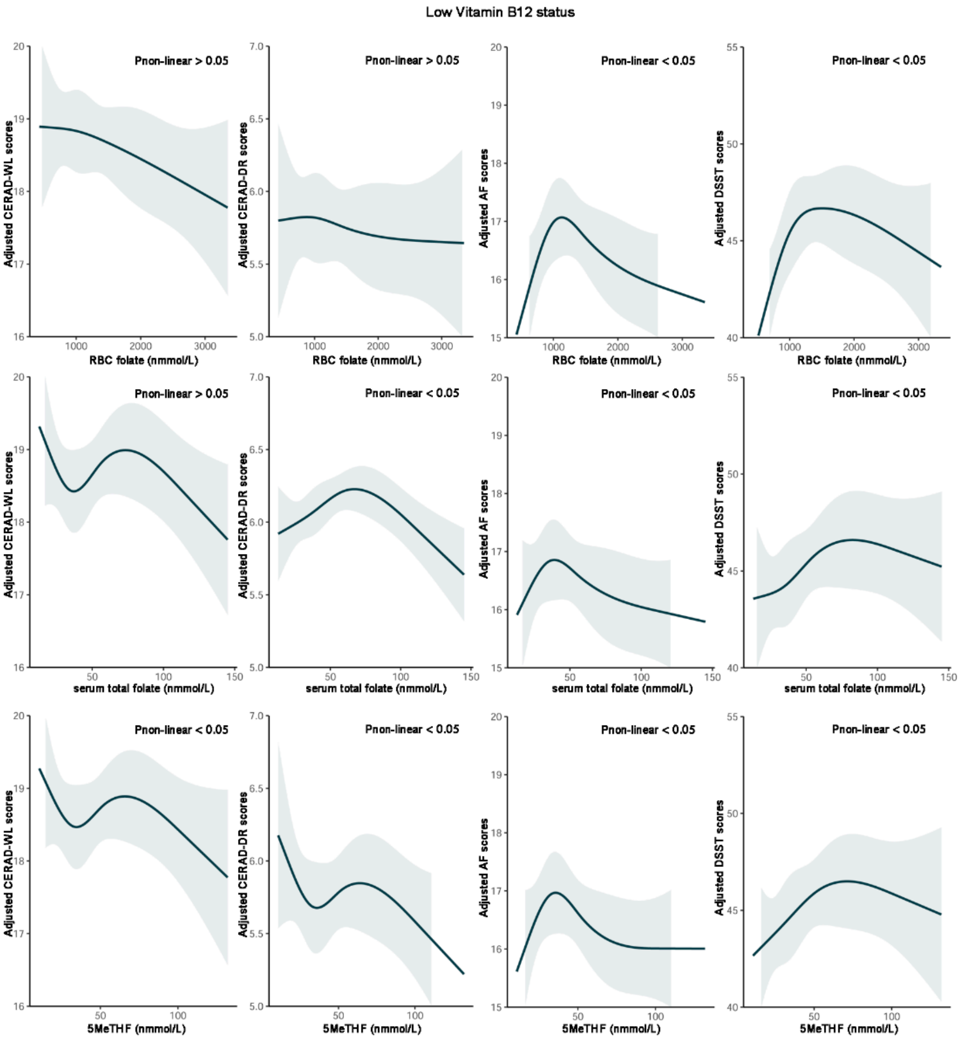

Figure S3. Association between folate status and cognitive performance in low vitamin B12 status. The association between cognitive level and RBC folate, serum total folate, 5MeTHF was assessed by restricted cubic spline curves based on linear model estimation using ordinary least squares. The solid black lines with shaded regions indicate the adjusted recognition test scores and their corresponding 95% confidence intervals, respectively.

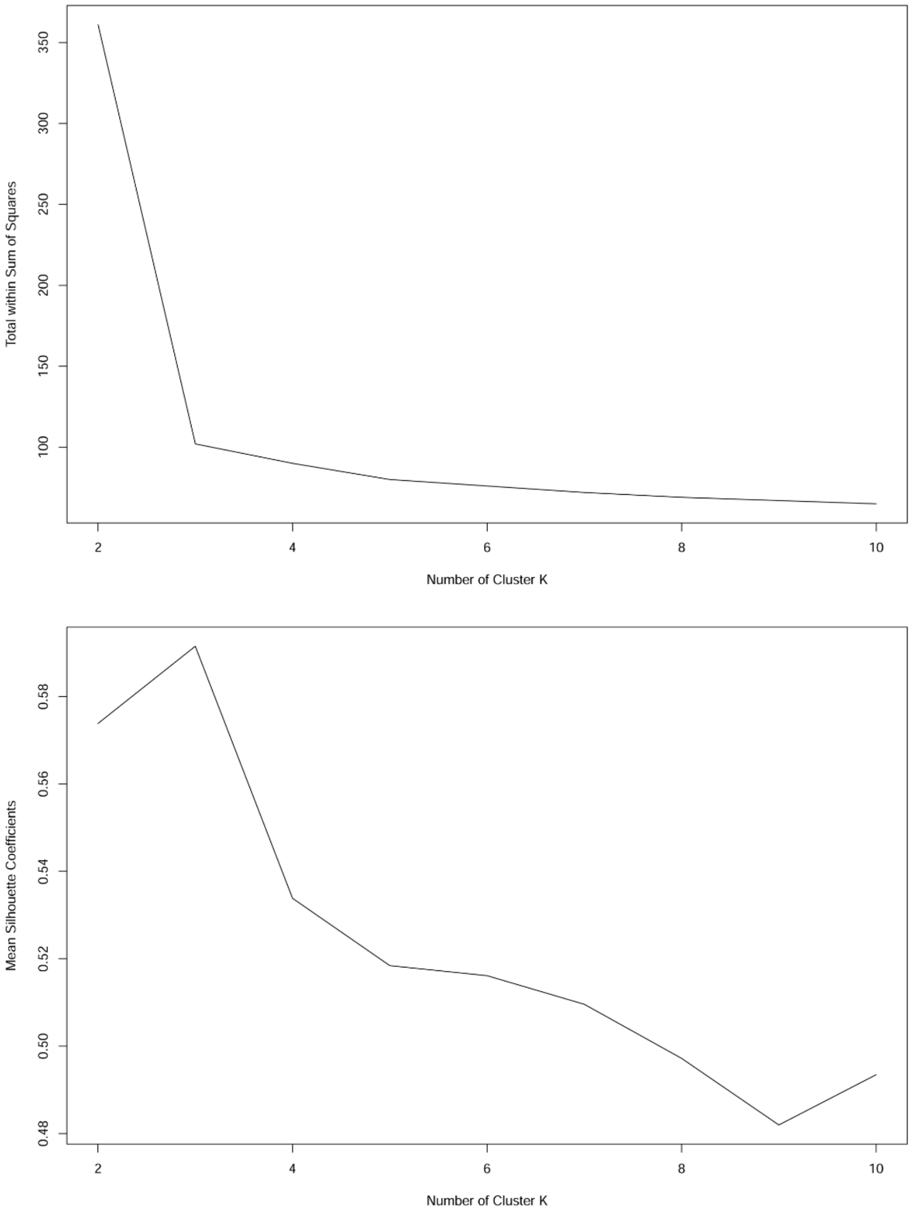

Figure S4. Elbow plot and Mean silhouette coefficients plot. Elbow plot and mean silhouette coefficients plot comparing cluster effect of different K. The elbow plot indicates the optimal number of clusters based on how tightly the clusters define their boundaries, and the mean silhouette coefficients define the compactness and separation of the clusters. When combining these 2 measures, one can estimate the optimal number of clusters, which in this case was determined to be 3.
